# After the program ends: HIV testing behavior among men who have sex with men after the conclusion of a program providing regular home delivery of HIV self-testing kits

**DOI:** 10.1186/s12879-025-10784-y

**Published:** 2025-04-03

**Authors:** Tyler B. Wray, Philip A. Chan, Jeffrey D. Klausner, Lori M. Ward, Erik M. S. Ocean

**Affiliations:** 1 Department of Behavioral and Social Sciences, Center for Alcohol and Addictions Studies, Brown University School of Public Health, 121 South Main Street, Providence, RI 02912 USA; 2 Department of Medicine, Warren Alpert Medical School of Brown University, Providence, RI USA; 3 Department of Population and Public Health Sciences, Keck School of Medicine, University of Southern California, Los Angeles, CA USA; 4 Department of Population Health Science, John D. Bower School of Population Health, University of Mississippi Medical Center, Jackson, MS USA

**Keywords:** HIV self-testing, Gay men, Men who have sex with men, Pre-exposure prophylaxis, Linkage to care, Telehealth

## Abstract

Testing for HIV continues to play a key role in prevention, especially among at-risk populations such as gay, bisexual, and other men who have sex with men (MSM). Incorporating HIV self-testing (HIVST) into testing programs encourages more frequent screening and likely facilitates earlier diagnosis, but little is known about how testing behavior changes after self-testing programs end. In this study, a subset of MSM in the eTest trial who were randomized to receive regular HIVST or clinic testing every three months for 12 months were followed for an additional 12 months. We compared testing rates during the intervention period to this 12-month post-intervention period. Results of a Poisson mixed model showed a significant condition by time interaction (IRR=0.91, SE=0.04, p=.019, %95CI=0.84-0.98). The average marginal predicted probability of testing in a given follow-up among controls was 30.4% during the intervention period and 28.2% post-intervention, versus 70.0% among HIVST condition participants during the intervention period but 23.6% by the end of the post-intervention period. Although regular mail delivery of HIVST increased HIV testing considerably while tests are actively being delivered, testing rates declined to a level similar to those who had not received HIVST after regular delivery stops. These results suggest that regularly delivering HIVST does not encourage longer-term regular testing habits in MSM who typically test infrequently.

**Supplementary Information:**

The online version contains supplementary material available at 10.1186/s12879-025-10784-y.

## Introduction

HIV incidence has declined in the United States over the last few years, but not enough to reach the Ending the Epidemic (EHE) plan’s target of reducing new infections by 90% in 2030 [1]. Gay, bisexual, and other men who have sex with men (MSM) continue to be one of the populations most affected by HIV, accounting for 70% of new infections in 2021 [2]. Promoting more consistent use of effective prevention methods in these populations, such as pre-exposure prophylaxis (PrEP) and treatment-as-prevention (TaSP), would help achieve more robust declines in new infections among these populations [3]. However, routine testing and diagnosing all new cases of HIV as soon as possible is another key pillar of the EHE plan in part because it can facilitate TaSP and PrEP [4]. Given this, HIV testing plays a key role in prevention. The CDC currently recommends that MSM test for HIV at least once per year, but more frequently if they have multiple (i.e., > 1) sex partners [5]. Yet, evidence suggests only 50–69% of MSM test this often [6, 7], which can result in delayed HIV diagnoses [8].

Incorporating HIV self-testing (HIVST) into testing programs encourages more frequent screening and facilitates earlier diagnosis. Outcome data from the first year of the CDC’s TakeMeHome program shows that home-delivered HIVST can reach high-priority populations with testing and help diagnose new HIV cases [9]. Findings reported from the first year of the program showed that 24% of those who requested a test kit had never tested for HIV before, and of those who completed a follow-up survey and used the kit themselves, 1.9% reported a reactive result. Meta-analyses have also shown that HIVST increases testing relative to relying on clinic-based testing alone [10]. However, the effects of HIVST were relatively modest across the ten studies included in this analysis, increasing testing uptake by only 45% relative to control conditions. Our work recently extended these findings by showing that the effects of HIVST on testing behavior could depend on the delivery approach used [11]. In a pragmatic, longitudinal trial of MSM, we showed that delivering HIVST every three months without requiring participants to request each test kit yielded nearly doubled the rate of testing over reminders for clinic-based testing alone. For any test during the 12 month period, adjusted probabilities of testing were 89–91% in self-testing conditions, versus 57% among controls. Moreover, of the eight new cases of HIV diagnosed during the study, all but one (88%) was diagnosed starting with an HIVST, suggesting that automatically delivering HIVST through the mail at regular intervals may also facilitate earlier diagnosis relative to relying on in-person testing strategies alone.

Another potentially appealing aspect of regular mail delivery of self-testing kits is the possibility that these programs could support a longer-term change in testing behavior among participants. That is, delivering self-test kits to those who struggle to test as frequently as recommended could encourage more regular testing, even after the program ends. However, we are not aware of any studies published to date that explore whether and how testing behavior changes after self-testing programs end.

In this study, we evaluated these questions in a subset of participants from our recent pragmatic trial of free, regular HIVST delivery. In the original trial, MSM who tested infrequently were recruited from EHE jurisdictions in several areas and were provided with one of the following every 3 months for 12 months: [1] mailed HIVST kits with access to a 24-hour helpline (standard HST) [2], mailed HIVST kits with follow-up phone counseling within 24 h, and [3] reminders to get tested at a local clinic (control). This initial 12-month period was the active intervention period, and participants completed online follow-up surveys at 1, 4, 7, 10, and 12 months. At 12 months, we invited 150 participants to continue completing follow-up surveys every three months (at 15, 18, 21, and 24 months) for an additional year. In this study, we compared HIV testing behavior in these participants during the active intervention period (first 12 months) and the 12-month period following the intervention period (post-intervention period). In particular, we compared rates of any testing, repeat testing (testing > 1 time), and testing within a given 90 day follow-up period across these two periods and across participants assigned to an HIVST condition during the intervention period versus those simply provided reminders to test in-person. We also explored the types of tests participants used during these two time periods and across conditions. Results can inform the design of HIVST programs to optimize screening coverage.

## Methods

In the original trial, we recruited 811 MSM from several EHE jurisdictions in the Northeast (Boston), West (Los Angeles), and several areas in the South (larger cities in Louisiana and Florida, as well as the state of Mississippi). Full lists of recruitment areas are available elsewhere [11, 12]. Participants were recruited from January 2019 to April 2022. All procedures were approved by the Brown University Institutional Review Board and the primary and secondary outcomes of the original trial were pre-registered on ClinicalTrials.gov (NCT03654690). The analyses reported here are secondary analyses of the data collected in this trial.

### Recruitment

Participants who were eligible for the original trial were: [1] 18+ [2], self-reported assigned male at birth [3], HIV-negative or unknown status, and reported [4] not taking or prescribed PrEP for the past six months [5], (a) having engaged in condomless anal sex (CAS) with a partner who was not sexually exclusive, (b) currently having anal sex with an HIV-positive partner, or (c) having been diagnosed with an STI in the last 6 months, and [6] not having tested for HIV in the past 6 months. Eligible participants also [7] reported owning a smartphone and [8] having a residence where they could securely receive packages, and [9] resided in one of the eligible locations.

Participants were recruited primarily using paid digital ads on a variety of popular online platforms, including search websites (e.g., Google), social media (e.g., Instagram, Facebook), and gay-oriented dating apps (e.g., Grindr, Jack’d). Details of this recruitment campaign have been described elsewhere [13]. Interested users clicked through an ad and completed a brief online survey that assessed basic eligibility criteria. If eligible and interested, participants were then asked to provide their informed consent and register for the study. Before being considered formally enrolled, participants were asked to complete a brief phone call with staff to confirm their personal information.

### Experimental conditions

Once enrolled, participants were randomized to receive one of the following every three months over a 12-month period: [1] text message reminders to get tested for HIV at a local clinic (control) [2], free OraSure^®^ OraQuick Advance HIV 1/2 self-testing kits (OraSure^®^ Technologies, Bethlehem, PA, USA) with access to a 24 h helpline (standard HIVST), and [3] the same HIVST kits equipped with a sensor (Estimote, Inc., New York City, NY, USA) that alerted staff HIV test counselors to initiate phone counseling within 24 h of a test kit being opened (eTest). Participants were assigned to one of these three groups, 1:1:1, using a random number generator.

### Procedures

All participants were asked to complete online follow-up surveys at 1, 4, 7, 10, and 12 months. Follow-up surveys assessed HIV testing since the previous survey, including both clinic-based and home-based services, as well as the results of any HIVST they used. We did not explicitly instruct participants to use HIVST. Follow-up surveys were staggered to avoid inadvertently prompting participants to use HIVST kits they had been provided. After completing their 12 month surveys, the first 150 participants enrolled in the larger trial were asked to complete quarterly online follow-up surveys over an additional year (15, 18, 21, and 24 months). No interventions or HIVST were provided during this extended follow-up period. Participants were compensated up to $350 based on their completion of study procedures.

Those assigned to the control condition received text messages at baseline, 3, 6, and 9 months reminding them to get tested for HIV at a local clinic. Text messages included a link to the CDC’s HIV service locator widget [14], which they could use to find free testing near them.

Those assigned to the standard HIVST condition received kits with standard follow-up resources, which included OraSure’s free 24-hour hotline. HIVST kits sent to participants in the eTest condition were equipped with Bluetooth sensors that detected when kits were opened and triggered an email alert to study counselors to follow up with participants. Postcards were included in all HIVST kits that provided information about a variety of local resources, including HIV prevention, sexual health, general healthcare, mental health, and alcohol/drug treatment. All study materials were available in both English and Spanish and were provided in participants’ primary language preferences.

### Measures

**HIV testing.** Online follow-up surveys asked participants to report the total number of times they had been tested for HIV in the last 3 months. If they reported having tested, follow-up questions then asked them to report the date, location (e.g., primary care clinic, HIV testing clinic, community organization, community event, at home), type of sampling used (e.g., blood - finger prick, blood – venipuncture, oral swab) and what the results of each test were. Participants assigned to an HIVST condition during the first 12 months intervention phase of data collection (standard, eTest) were also asked whether they received a home test sent to them by the study since the last survey, and if so, whether they used it to test themselves in the past 3 months and what the results were. During the extended, 12-month post-intervention follow-up period, all participants were asked the same questions regardless of previously assigned condition. Participants who reported testing at a clinic were asked to sign a release that allowed staff to verify these responses.

### Statistical analysis

The focal outcomes in this study were three binary variables: [1] whether participants reported takingat least one HIV test on any follow-up survey within each 12-month period in the study (intervention [0–12 months], post-intervention [12–24 months]) (*any* HIV testing) [2], *repeat* HIV testing, or whether participants reported taking > 1 test on any follow-up survey within each 12-month period, and [3] reporting an HIV test in a given quarterly follow-up survey. Each of these outcomes compared those that met these conditions to those who were enrolled but did not test (eligible to be tested). Although participants reported the total number of HIV tests they took on follow-up surveys, we elected to model the above binary variables as opposed to count data because of very high skewness in tests reported and the additional modeling complexity that would be required to analyze a count outcome. Since we found few differences between the standard HIVST and eTest groups in these outcomes in our main outcomes analysis, we collapsed these two groups in the present study, creating a single group that reflected participants who received HIVST every three months during the 12-month intervention period. We first estimated rates of any and repeat testing during the intervention phase by condition and compared these unadjusted rates to those in the 12-month post-intervention period using basic Chi-square statistics. We then compared various types of tests participants used within each group across the 12-month intervention period and the 12-month post-intervention period. Next, we plotted the total percentage of tests taken by type across the entire 24-month period by condition to show which types of testing participants in each condition adopted after the intervention phase. Finally, to estimate the probability of testing in each quarter by condition during each phase, we fit a Poisson mixed model with HIV testing in each quarter as an outcome and random intercepts. We used Poisson regression given that HIV testing was common in our sample and logistic models can produce inflated odds ratios (ORs) when the outcome is common. As in our main outcomes study [11], we controlled for participants’ baseline frequency of testing by including a categorical variable reflecting whether participants had reported being tested for HIV at least once per year over the three years prior to enrolling or not. We also included a two-way interaction between intervention-phase condition assignment (HIVST group vs. control) and follow-up month. In this model, we used an unstructured correlation matrix, requested robust standard errors, and used predictive margins after estimation to estimate the probability of testing in each condition and time period. We elected to fit a mixed model as opposed to alternatives (e.g., generalized estimating equation) because of its ability to handle unbalanced data without requiring assumptions about the missing data mechanism. As in our primary outcomes study, we adopted a conservative approach to missing data by assuming that all missing follow-up surveys reflected no testing. Analyses were performed in Stata SE 16 (College Station, TX, USA). The study was approved by the Brown University institutional review board.

## Results

See Table 1 for demographics of the analyzed subset of participants. A total of 150 participants were invited to complete follow-up surveys for an additional 12 months after the 12-month post-intervention period. Of those invited, 134 participants (89.3%) completed at least one survey after the 12-month intervention period. Nearly half of participants completed all four assigned follow-ups during the post-intervention period (49.3%), 16.7% completed three, 16.7% completed two, and 19.4% completed one. Participants included in this subsample were on average in their mid-thirties (*M* = 35.0, *SD* = 13.6), with 87.3% identifying as either gay or bisexual, and 43.3% were Black or Hispanic/Latino. 55% also tested less frequently than at least once per year in the three years before enrolling in the study.

**Table 1:** Demographic and behavioral characteristics of the analyzed sample (*N* = 134)

Characteristics	All (*N* = 134)	Control (*N* = 37)	HIVST (*N* = 97)
	Mean (SD) or *N* (%)	Mean (SD) or *N* (%)	Mean (SD) or *N* (%)
Age (Range: 18–78, *M* ± SD)	35.0 (13.6)	33.9 (12.1)	35.4 (14.1)
Trans/other gender identity	1 (0.8)	0 (0.0)	1 (1.0)
Race			
White	84 (62.7)	24 (64.9)	60 (61.9)
Black or African American	15 (11.2)	2 (5.4)	13 (13.4)
American Indian/Alaska Native	1 (0.8)	0 (0.0)	1 (1.0)
Asian	7 (5.2)	3 (8.1)	4 (4.1)
Multiracial	9 (6.7)	3 (8.1)	6 (6.2)
Chose not to respond	18 (13.4)	5 (13.5)	13 (13.4)
Ethnicity (Hispanic or Latino)	45 (33.6)	15 (40.5)	30 (30.9)
Spanish primary language	5 (3.7)	1 (2.7)	4 (4.1)
Single relationship status	78 (58.2)	19 (51.4)	59 (60.8)
College degree	70 (52.2)	20 (54.1)	50 (51.6)
Low income^1^	41 (30.6)	10 (27.0)	31 (32.0)
Unemployed	19 (14.2)	5 (13.5)	14 (14.4)
Gay or bisexual identity	117 (87.3)	33 (89.2)	84 (86.6)
Region			
Northeast	49 (36.6)	15 (40.5)	34 (35.1)
South	14 (10.4)	3 (8.1)	11 (11.3)
West	71 (53.0)	19 (51.4)	52 (53.6)
Never tested for HIV, lifetime	15 (11.1)	6 (16.2)	9 (9.3)
Last HIV test, in years	2.6 (5.2)	1.2 (1.0)	3.1 (6.0)
< 1 HIV test/year, past 3 years			
CAS with high-risk partner^2^	129 (96.3)	35 (94.6)	94 (94.6)
Regular sex w/ HIV-positive partner^2^	12 (9.0)	4 (10.8)	8 (8.3)
Diagnosed w/ STI	15 (11.2)	2 (5.4)	13 (13.4)
Ever had PrEP prescription	19 (14.2)	7 (18.9)	12 (12.4)

Note. All questions referred to the past 12 months. ^1^Represents those with a household income <$30,000 a year. ^2^CAS=Condomless anal sex. STI = sexually transmitted infection. Participants must have met one or more of these criteria for PrEP eligibility to enroll

93% of this subsample reported testing at least once during the 12-month intervention period, including 99.0% of participants assigned to an HIVST condition and 75.7% assigned to control (χ^2^[1] = 21.0, *p* <.001). Only 72.4% of all participants reported testing for HIV at least once during the 12 months following the intervention period, including 76.3% of HIVST participants and 62.2% of control participants (χ^2^[1] = 2.7, *p* =.102). Similarly, 82.1% of all participants reported repeat testing (> 1 test) during the intervention period, including 94.9% of HIVST participants and 48.7% of controls (χ^2^[1] = 38.9, *p* <.001). During the post-intervention period, only 38.8% of participants reported repeat testing, including 42.3% of those in an HIVST condition and 29.7% of controls (χ^2^[1] = 1.8, *p* =.183). See Table 2 for unadjusted estimates by condition.

**Table 2:** Unadjusted rates of any testing and repeat testing (> 1 test) during the 12-month intervention period and 12-month post-intervention period by condition

Characteristics	Intervention period		Post-intervention period	
	Control (*N* = 37)	HIVST (*N* = 97)	Control (*N* = 37)	HIVST (*N* = 97)
	*N* (%)	*N* (%)	*N* (%)	*N* (%)
Any testing (*≥* 1 test in 12 mo.)	28 (75.7)	96 (99.0)	23 (62.2)	74 (76.3)
Repeat testing (> 1 test in 12 mo.)	18 (48.7)	92 (94.9)	11 (29.7)	41 (42.3)
*Test types used* ^*1*^				
In-person testing only	23 (85.2)	1 (1.0)	13 (56.5)	16 (21.6)
HIVST (independently purchased) only	3 (11.1)	0 (0.0)	9 (39.1)	4 (5.4)
HIVST (study-provided) only	0 (0.0)	39 (40.6)	0 (0.0)	34 (46.0)
In-person & purchased HIVST	1 (3.7)	0 (0.0)	1 (4.4)	3 (4.1)
In-person & study-provided HIVST	0 (0.0)	45 (46.9)	0 (0.0)	16 (21.6)
Purchased & study-provided HIVST	0 (0.0)	4 (4.2)	0 (0.0)	1 (1.4)
All three types	0 (0.0)	7 (7.3)	0 (0.0)	0 (0.0)
*Locations of in-person tests used* ^*2*^				
Primary care doctor	22 (42.3)	31 (50.8)	9 (40.9)	21 (41.2)
HIV testing clinic	21 (40.3)	18 (29.5)	11 (50.0)	13 (25.4)
Community event	6 (11.5)	6 (9.8)	2 (9.1)	12 (23.5)
Other medical clinic	3 (5.8)	6 (9.8)	0 (0.0)	5 (9.8)
Other	0 (0.0)	0 (0.0)	0 (0.0)	0 (0.0)

Note: ^1^Includes only those who reported any testing. ^2^Includes only in-person tests that were reported and reflects the percent of all reported in-person tests. Participants could report having been tested at multiple in-person locations

**Fig. 1:**
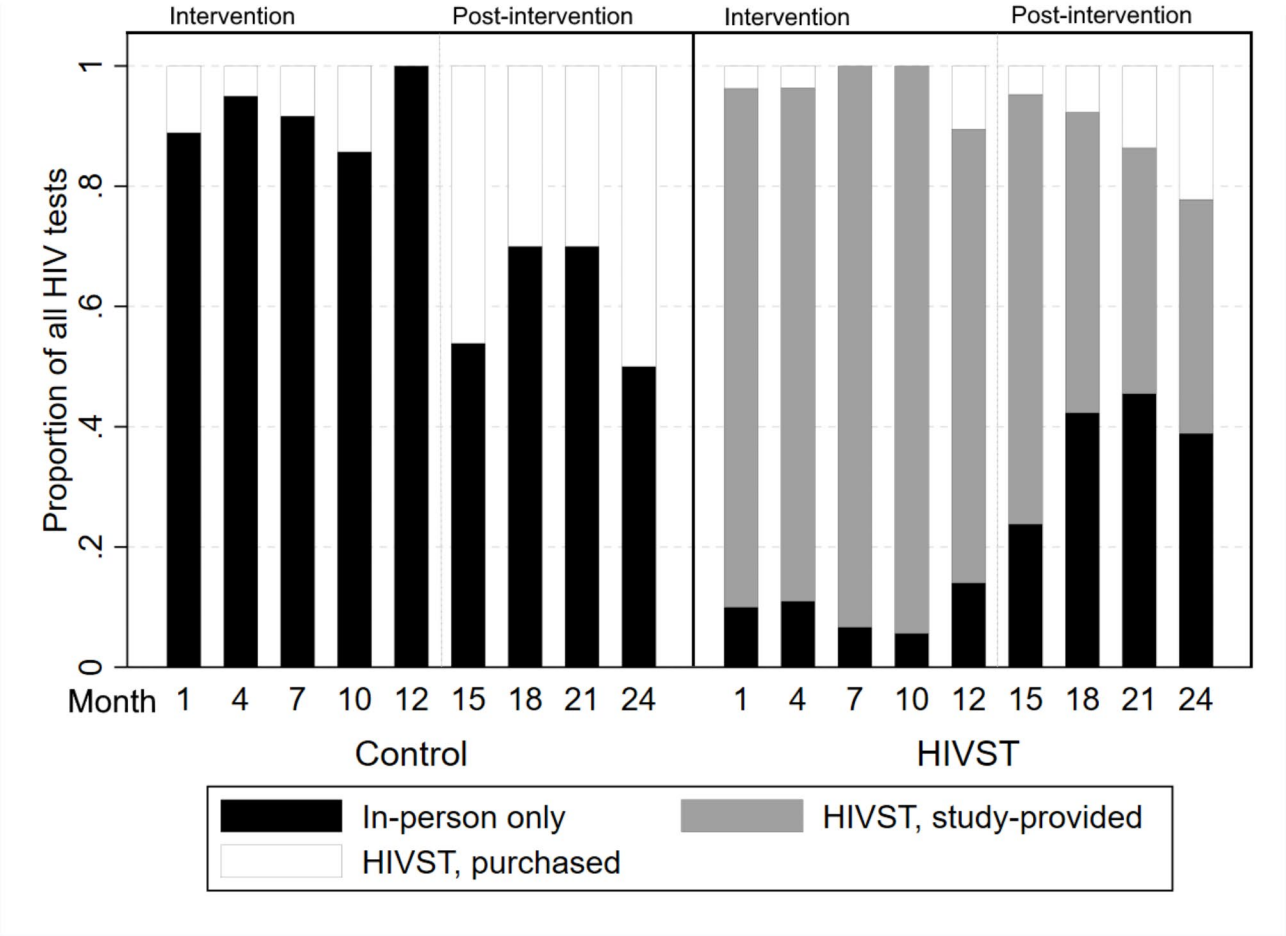
Proportions of types of HIV tests used at each follow-up period by condition assignment across the intervention period (first 12 months) and post-intervention period (second 12 months)

Among control participants who tested for HIV during the intervention period, 85.2% were tested only in-person, 11.1% used HIVSTs not provided by the study, and 3.7% both tested in-person and through HIVST. During the post-intervention period, 56.5% of control participants who tested were tested in-person, 39.1% used HIVSTs, and 4.4% were tested in-person and used HIVSTs. Among those in an HIVST condition who tested during the intervention period, 46.9% tested both in-person and through study-provided HIVSTs, 40.6% tested using study-provided HIVSTs only, 7.3% tested in-person and used HIVSTs both provided by the study and not provided by the study, and 4.2% used HIVSTs both provided by the study and not provided by the study. During the post-intervention period, 46.0% of participants assigned to an HIVST condition tested using only study-provided HIVSTs, 21.6% tested only in-person, 21.6% tested using both study-provided HIVSTs and in-person tests, 5.4% used only HIVSTs that were not study-provided, 4.1% tested via both in-person and HIVSTs not provided by the study, and 1.4% tested via both purchased and study-provided HIVST. See Fig. 1 for the proportions of testers who used each type of test at each follow-up timepoint by condition.

**Fig. 2:**
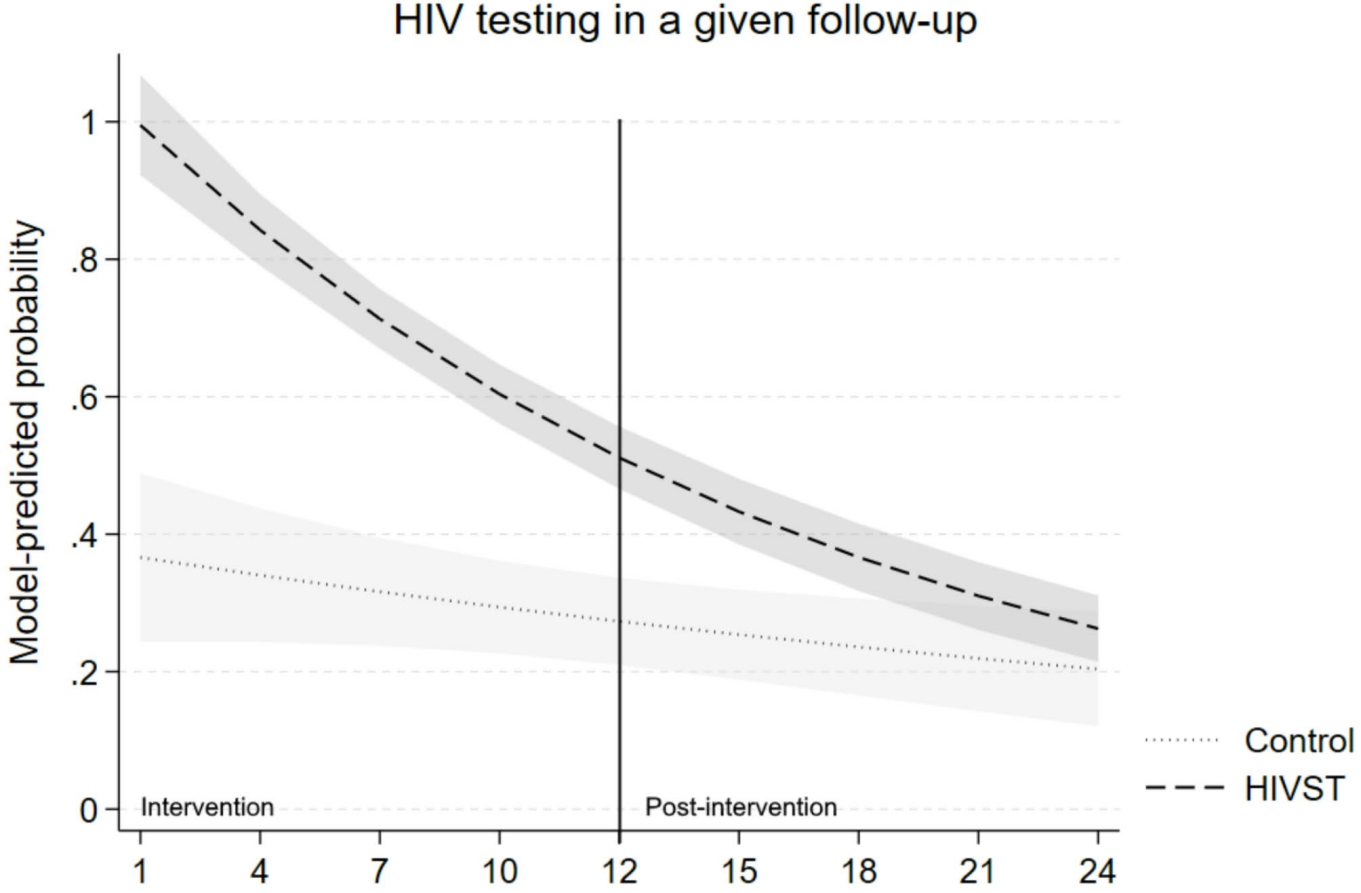
Model-predicted probabilities of testing for HIV in a given follow-up period over the intervention period (first 12 months) and the post-intervention period (second 12 months)

In this subsample, participants reported having tested during 48.1% of assessed follow-ups, including 56.1% of follow-ups in participants assigned to the HIVST condition and 27.6% of follow-ups among those in the control condition. In the mixed-effects Poisson regression model (*N* = 138 participants, across T = 9 follow-up periods, for a total of NT = 1,242 observations), the interaction between condition assignment and follow-up period was statistically significant (IRR = 0.91, 95% CI: 0.84, 0.98, *p* =.019), indicating that the probability of testing declined more steeply over time in the HIVST group compared to the control group (see Table 3). The predicted probability of testing in the control group was 36.6% at the first follow-up, declining to 20.4% by the final follow-up (see Fig. 2). In contrast, the HIVST group started at 99.5% in the first follow-up but declined to 26.3% by the final follow-up. These findings suggest that while participants in the HIVST condition were significantly more likely to test throughout the study period (IRR = 2.98, 95% CI: 2.00, 4.45, *p* <.001), this effect diminished over time as testing rates in both groups declined.

**Table 3:** Poisson mixed model of HIV testing in a given quarter over the 24-month study period

Variable	Any HIV testing			
	IRR	SE	*p*	%95 CI
< 1 test/yr, past 3 yrs^1^	0.91	0.06	0.172	0.79–1.04
HIVST condition	2.98	0.61	< 0.001	2.00-4.45
Follow-up period	0.93	0.03	0.049	0.86-1.00
HIVST condition * follow- up period	0.91	0.04	0.019	0.84–0.98

Note. ^1^Dummy variable representing whether participants reported testing at least three times in the three years prior to enrolling in the study

## Discussion

The results of this study lend important insights about the HIV testing behavior of at-risk MSM after they stop receiving regularly-delivered, free self-testing kits. Overall, while providing free HIVST kits through the mail at regular intervals appears to encourage substantial changes in testing behavior during the program, the probability of testing generally decreases to a similar rate of controls after HIVST intervention. That is, regularly delivering free HIVST over a year does not appear to promote lasting changes in testing behavior in MSM who test infrequently. Both unadjusted proportions and results of a longitudinal model of the odds of testing during a given follow-up interval confirm these findings and show substantial differences across HIVST and control conditions during the intervention period, but much smaller or no differences in the odds or probability of testing during the post-intervention period. The longitudinal model, in particular, suggested that, though pairwise comparisons between these two conditions were significant in months 1–18, by months 18–24, the 95% confidence intervals around the estimated probability of testing across the two groups overlapped, suggesting no significant differences between the groups beyond that point.

Our findings also suggest that much of the difference in the probability of testing across conditions during the post-intervention period are likely due to participants who were assigned to the HIVST group saving their test kits from the intervention period to use after the program stopped. Although participants assigned to an HIVST group appeared to gradually test more in-person as the post-intervention period went on, even in months 21–24, about 40% of participants who tested for HIV reported doing so using a study-provided HIVST. These findings support the conclusion that MSM who receive regular home delivery of HIVST are likely to return to testing in ways that are similar to controls after the program is completed. Cost is one factor that could explain this pattern of findings. Since few other programs were providing free HIVST at the time this sample was recruited, participants would have likely needed to purchase HIVST kits at retail locations in order to continue using it after this study intervention period finished. This would likely be a significant barrier to continued use of HIVST, particularly given that clinic-based testing can often be accessed for free or low cost in many areas of the US. This is consistent with past research showing that cost is a significant barrier to HIVST use across many populations [15, 16].

Other notable results showed that nearly a third of participants assigned to receive regular deliveries of HIVSTs tested in-person at least once during the intervention period, while they were also receiving test kits. Given low uptake of PrEP in the primary study [11], it is unlikely that this finding simply reflects increased in-person testing related to PrEP care. Instead, this result could suggest that some participants continue to test in-person while also using HIVST, perhaps at the recommendation of their providers. However, it could also reflect some initial uncertainty about the reliability of HIVST, especially early on in the study. This latter possibility is consistent with other findings that adoption of non-study-provided HIVST gradually increased among control participants over the course of the study period. It is likely that this reflects natural adoption of HIVST over time as it became a more legitimate option for screening. As follow-ups in this subsample were collected from 2019 to 2021, these findings would be consistent with increasing familiarity and acceptance of home testing generally because of COVID home testing and other programs being offered during that period.

### Limitations

This study has important strengths, but several limitations are also important to note. First, the subsample included in these analyses consisted of only 134 total participants, which is a relatively small subset (16%) of the sample recruited in the full study. Although these participants completed over 1,000 follow-ups and yielded convincing estimates of all effects, it is possible that the results reported here may have been different had a larger sample been recruited for this extended portion of the study. Second, this subsample involved a relatively low percentage of participants from areas of the US South relative to the full trial (10%) [11], likely because these participants were recruited relatively early during the full trial when our recruitment operations in the South were underperforming. It is possible that the reported results may have been different had our subsample represented more MSM living in areas of the US South. A similar limitation extends to inclusion of racial and ethnic minority MSM in this subsample, although the percentage of participants who identified as Black/African American or Hispanic/Latino was relatively high overall (43.3%). Third, the outcomes reported in this manuscript were based on self-report and thus could be subject to demand effects or bias relative to more objective approaches to assessment (e.g., medical records). However, participants were informed that our team would verify reports of clinical services and were asked to authorize their providers/clinics to release records of testing to us, which may have reduced reporting inaccuracies.

## Conclusions

In summary, we found that, although regular mail delivery of HIVST increased HIV testing considerably during a 12-month intervention period, testing rates declined to a level that was similar to MSM who had not received HIVST during the 12-month period after the program ended. These results suggest that regularly delivering HIVST does not encourage longer-term regular testing habits in MSM who typically test infrequently. For optimal testing coverage and early detection, HIVST program planners should ideally identify those in especially high-risk life periods and deliver testing throughout these periods, which previous research suggests are often around two years [17]. Future research should focus on determining intervals to provide HIVST that are optimal for encouraging testing and diagnosing new HIV cases earlier during periods of increased risk.

## Electronic supplementary material

Below is the link to the electronic supplementary material.

Supplementary Material 1

Supplementary Material 2

## Data Availability

Deidentified data that support the findings of this study are available from the corresponding author upon reasonable request.

## Declarations

### Clinical trial

Number: NCT03654690; Registration Date: 01/23/2019.

## Abbreviations

EHE: Ending the HIV Epidemic
HIV: Human immunodeficiency virus
HIVST: HIV self-testing
PrEP: Pre-exposure prophylaxis
MSM: Men who have sex with men
STI: Sexually transmitted infection
US: United States
