# Correction: After the program ends: HIV testing behavior among men who have sex with men after the conclusion of a program providing regular home delivery of HIV self-testing kits

**DOI:** 10.1186/s12879-025-11042-x

**Published:** 2025-05-05

**Authors:** Tyler B. Wray, Philip A. Chan, Jeffrey D. Klausner, Lori M. Ward, Erik M. S. Ocean

**Affiliations:** 1 Department of Behavioral and Social Sciences, Center for Alcohol and Addictions Studies, Brown University School of Public Health, 121 South Main Street, Providence, RI 02912 USA; 2 Department of Medicine, Warren Alpert Medical School of Brown University, Providence, RI USA; 3 Department of Population and Public Health Sciences, Keck School of Medicine, University of Southern California, Los Angeles, CA USA; 4 Department of Population Health Science, John D. Bower School of Population Health, University of Mississippi Medical Center, Jackson, MS USA

**Correction: Wray et al. BMC Infectious Diseases (2025) 25:462**

10.1186/s12879-025-10784-y

Following publication of the original article [1], we were notified of an error in the article’s title.

Originally published title: After the program ends: HIV testing behavior among men who have sex **when** men after the conclusion of a program providing regular home delivery of HIV self-testing kits.

Corrected title: After the program ends: HIV testing behavior among men who have sex **with** men after the conclusion of a program providing regular home delivery of HIV self-testing kits.

The original article has been corrected.
