# Supplementary material for: After the program ends: HIV testing behavior among men who have sex with men after the conclusion of a program providing regular home delivery of HIV self-testing kits
Source: BMC Infect Dis. 2025 Apr 3;25:462. doi: 10.1186/s12879-025-10784-y (PMC11969819; doi:10.1186/s12879-025-10784-y)
Supplement: Supplementary file 1 — Supplementary Material 1 [file 12879_2025_10784_MOESM1_ESM.docx]

eTest – Quarterly Follow-Up

* We developed these measures specifically for this follow-up study “After HST”.

Q56 How many times have you been tested for HIV **since the last study survey you took (approximately ${e://Field/monthssincesurvey} month(s) ago)**? Do not include any home tests we sent you as part of the study. We'll ask you more about that later.

________________________________________________________________

Q57 On what date were you last tested for HIV? Enter an approximate date in MM/DD/YYYY format (e.g., 07/01/2018). If you're unsure, provide your best guess. Again, do not include any home tests we sent you as part of the study.

________________________________________________________________

Q58 Where were you **last**tested for HIV **in the past ${e://Field/**monthssincesurvey**} month(s)**? Check all that apply. Again, do not include any home tests we sent you as part of the study.

- Your primary care doctor's office
- HIV testing clinic
- Other medical clinic
- Community event
- At home
- Other __________________________________________________

| 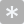 |
| --- |

Q59 You said you last tested for HIV at ${Q58/ChoiceGroup/SelectedChoices}. Type the name of the clinic, agency, or doctor’s office(s) where you were tested for HIV as carefully and accurately as you can. For example, if you got tested at your primary care doctor’s office, type “Dr. Doe’s office.”

________________________________________________________________

________________________________________________________________

________________________________________________________________

________________________________________________________________

________________________________________________________________

Q156 Below are more questions regarding the clinic, agency, or doctor's office where you were last tested for HIV. Please complete as much of the information below as possible.

- Office/Clinic name: __________________________________________________
- Doctor's full name: __________________________________________________
- Office/Clinic phone number: __________________________________________________
- Office/Clinic address: __________________________________________________

Q60 Was this HIV test associated with PrEP care (i.e. an appointment with the doctor who gives you your PrEP prescription, if you are on PrEP).

- No
- Yes

Q61 What were the results of the last HIV test you took **in the past ${e://Field/monthssincesurvey} month(s)**?

- Negative
- "Reactive" or preliminary positive
- Inconclusive

Q62 Have you already contacted a physician or clinic to schedule an appointment for confirmatory testing or follow-up?

- No
- Yes

Q64 Thanks for your honesty. A counselor from the study team will be following up with you over the phone in the next few hours, at the phone number you provided when you joined the study. He/she will ask you some more questions about your test and help you with the next steps. In the meantime, you can finish the survey.   As always, feel free to call us anytime (24-hours a day) at 401-863-9424 or send us an email (etest@brown.edu) with any urgent questions or concerns.

Q65 Did you receive a home-based HIV test mailed to you by this study **since you last completed a study survey, ${e://Field/monthssincesurvey} month(s) ago**?

- No
- Yes

Q66 Did you use the home-based HIV test that was provided to you by this study to test yourself for HIV **sometime in the past ${e://Field/monthssincesurvey} month(s)**?

- No
- Yes

Q146 What were the results of this home-based HIV test that was provided to you by this study?

- Negative
- "Reactive" or preliminary positive
- Inconclusive

Q147 Have you already contacted a physician or clinic to schedule an appointment for confirmatory testing or follow-up?

- No
- Yes

Q148 Thanks for your honesty. A counselor from the study team will be following up with you over the phone in the next few hours, at the phone number you provided when you joined the study. He/she will ask you some more questions about your test and help you with the next steps. In the meantime, you can finish the survey.   As always, feel free to call us anytime (24-hours a day) at 401-863-9424 or send us an email (etest@brown.edu) with any urgent questions or concerns.

Q67 Did you use the home HIV test we mailed you to test someone else (i.e., a friend, family member, potential partner)?

- No
- Yes

Q68 Did you give the home HIV test we mailed you away to someone else?

- No
- Yes

Q69 Who did you test or give the home HIV test we mailed you to?

- A friend
- An acquaintance
- A family member
- Someone I was having/had sex with, or was interested in having sex with
- Work colleague
- I sold it
- I lost it
- Other __________________________________________________

Q149 In the past 3 months, have you received a reactive ('preliminary positive') or inconclusive/invalid test result when taking a home-based HIV test mailed to you as part of this study (the eTest study)?

- No
- Yes
- I have not received any home-based HIV tests as part of this study.
- I have not taken any home-based HIV tests sent to me as part of this study.
